# Supplementary material for: Role and mechanism of NCAPD3 in promoting malignant behaviors in gastric cancer
Source: Front Pharmacol. 2024 Apr 22;15:1341039. doi: 10.3389/fphar.2024.1341039 (PMC11070777; doi:10.3389/fphar.2024.1341039)
Supplement: Supplementary file 11 [file DataSheet2.ZIP › GSEA/GO_CC/my_analysis.Gsea.1599461408869/GO_RIBONUCLEOPROTEIN_COMPLEX.html]

Details for gene set GO\_RIBONUCLEOPROTEIN\_COMPLEX[GSEA]

|  || Dataset | filtered\_dataset.sample\_info.cls#WT\_versus\_NCAPD3\_MUT |
| Phenotype | sample\_info.cls#WT\_versus\_NCAPD3\_MUT |
| Upregulated in class | WT |
| GeneSet | GO\_RIBONUCLEOPROTEIN\_COMPLEX |
| Enrichment Score (ES) | 0.35419944 |
| Normalized Enrichment Score (NES) | 2.291873 |
| Nominal p-value | 0.0 |
| FDR q-value | 0.0072078574 |
| FWER p-Value | 0.009 |
Table: GSEA Results Summary

  

Fig 1: Enrichment plot: GO\_RIBONUCLEOPROTEIN\_COMPLEX      
 Profile of the Running ES Score & Positions of GeneSet Members on the Rank Ordered List

  

| SYMBOL | TITLE | RANK IN GENE LIST | RANK METRIC SCORE | RUNNING ES | CORE ENRICHMENT || 1 | 8568 | RRP1 | 22 | 1.027 | 0.0138 | Yes |
| 2 | 11052 | CPSF6 | 89 | 0.814 | -0.0117 | Yes |
| 3 | 55781 | RIOK2 | 135 | 0.763 | -0.0230 | Yes |
| 4 | 23450 | SF3B3 | 178 | 0.697 | -0.0339 | Yes |
| 5 | 51018 | RRP15 | 192 | 0.685 | -0.0235 | Yes |
| 6 | 6191 | RPS4X | 196 | 0.682 | -0.0057 | Yes |
| 7 | 3303 | HSPA1A | 206 | 0.669 | 0.0073 | Yes |
| 8 | 3304 | HSPA1B | 207 | 0.669 | 0.0270 | Yes |
| 9 | 55599 | RNPC3 | 227 | 0.652 | 0.0320 | Yes |
| 10 | 91574 | C12orf65 | 238 | 0.646 | 0.0435 | Yes |
| 11 | 6426 | SRSF1 | 243 | 0.637 | 0.0592 | Yes |
| 12 | 87178 | PNPT1 | 263 | 0.622 | 0.0633 | Yes |
| 13 | 9724 | UTP14C | 301 | 0.591 | 0.0530 | Yes |
| 14 | 129831 | RBM45 | 310 | 0.587 | 0.0643 | Yes |
| 15 | 84946 | LTV1 | 319 | 0.583 | 0.0754 | Yes |
| 16 | 26156 | RSL1D1 | 332 | 0.574 | 0.0834 | Yes |
| 17 | 5356 | PLRG1 | 338 | 0.573 | 0.0965 | Yes |
| 18 | 10885 | WDR3 | 430 | 0.515 | 0.0435 | Yes |
| 19 | 54931 | TRMT10C | 446 | 0.507 | 0.0472 | Yes |
| 20 | 8607 | RUVBL1 | 451 | 0.506 | 0.0591 | Yes |
| 21 | 27340 | UTP20 | 455 | 0.501 | 0.0715 | Yes |
| 22 | 10291 | SF3A1 | 466 | 0.493 | 0.0786 | Yes |
| 23 | 10969 | EBNA1BP2 | 483 | 0.485 | 0.0809 | Yes |
| 24 | 23468 | CBX5 | 484 | 0.485 | 0.0952 | Yes |
| 25 | 84261 | FBXW9 | 491 | 0.477 | 0.1047 | Yes |
| 26 | 10940 | POP1 | 497 | 0.474 | 0.1149 | Yes |
| 27 | 3184 | HNRNPD | 508 | 0.470 | 0.1213 | Yes |
| 28 | 1660 | DHX9 | 510 | 0.469 | 0.1343 | Yes |
| 29 | 84081 | NSRP1 | 538 | 0.453 | 0.1274 | Yes |
| 30 | 3609 | ILF3 | 544 | 0.449 | 0.1369 | Yes |
| 31 | 9416 | DDX23 | 553 | 0.445 | 0.1440 | Yes |
| 32 | 78988 | MRPL57 | 557 | 0.441 | 0.1548 | Yes |
| 33 | 6632 | SNRPD1 | 563 | 0.440 | 0.1640 | Yes |
| 34 | 51388 | NIP7 | 593 | 0.428 | 0.1549 | Yes |
| 35 | 6208 | RPS14 | 604 | 0.421 | 0.1597 | Yes |
| 36 | 10445 | MCRS1 | 617 | 0.414 | 0.1629 | Yes |
| 37 | 54663 | WDR74 | 628 | 0.408 | 0.1674 | Yes |
| 38 | 26747 | NUFIP1 | 666 | 0.394 | 0.1513 | Yes |
| 39 | 23212 | RRS1 | 681 | 0.387 | 0.1522 | Yes |
| 40 | 10607 | TBL3 | 698 | 0.380 | 0.1514 | Yes |
| 41 | 23076 | RRP1B | 709 | 0.370 | 0.1548 | Yes |
| 42 | 6182 | MRPL12 | 729 | 0.359 | 0.1512 | Yes |
| 43 | 55759 | WDR12 | 731 | 0.359 | 0.1610 | Yes |
| 44 | 134430 | WDR36 | 733 | 0.359 | 0.1708 | Yes |
| 45 | 58509 | CACTIN | 748 | 0.351 | 0.1707 | Yes |
| 46 | 51602 | NOP58 | 750 | 0.348 | 0.1802 | Yes |
| 47 | 440275 | EIF2AK4 | 753 | 0.347 | 0.1889 | Yes |
| 48 | 27316 | RBMX | 762 | 0.342 | 0.1929 | Yes |
| 49 | 494115 | RBMXL1 | 763 | 0.342 | 0.2030 | Yes |
| 50 | 3188 | HNRNPH2 | 769 | 0.338 | 0.2092 | Yes |
| 51 | 4869 | NPM1 | 772 | 0.336 | 0.2176 | Yes |
| 52 | 51118 | UTP11L | 773 | 0.336 | 0.2275 | Yes |
| 53 | 10492 | SYNCRIP | 779 | 0.334 | 0.2336 | Yes |
| 54 | 4670 | HNRNPM | 790 | 0.330 | 0.2358 | Yes |
| 55 | 1736 | DKC1 | 791 | 0.330 | 0.2455 | Yes |
| 56 | 51154 | MRTO4 | 794 | 0.327 | 0.2537 | Yes |
| 57 | 23246 | BOP1 | 797 | 0.325 | 0.2617 | Yes |
| 58 | 79590 | MRPL24 | 800 | 0.320 | 0.2697 | Yes |
| 59 | 27341 | RRP7A | 801 | 0.320 | 0.2791 | Yes |
| 60 | 91695 | RRP7BP | 802 | 0.320 | 0.2885 | Yes |
| 61 | 9221 | NOLC1 | 816 | 0.307 | 0.2878 | Yes |
| 62 | 51373 | MRPS17 | 817 | 0.306 | 0.2968 | Yes |
| 63 | 9343 | EFTUD2 | 818 | 0.305 | 0.3058 | Yes |
| 64 | 26121 | PRPF31 | 825 | 0.301 | 0.3101 | Yes |
| 65 | 11157 | LSM6 | 830 | 0.296 | 0.3159 | Yes |
| 66 | 10528 | NOP56 | 837 | 0.285 | 0.3198 | Yes |
| 67 | 6183 | MRPS12 | 838 | 0.280 | 0.3280 | Yes |
| 68 | 23435 | TARDBP | 842 | 0.272 | 0.3338 | Yes |
| 69 | 4691 | NCL | 843 | 0.270 | 0.3417 | Yes |
| 70 | 10856 | RUVBL2 | 847 | 0.257 | 0.3470 | Yes |
| 71 | 3312 | HSPA8 | 848 | 0.244 | 0.3542 | Yes |
| 72 | 8780 | RIOK3 | 948 | -0.367 | 0.2908 | No |
| 73 | 80336 | PABPC1L | 971 | -0.387 | 0.2858 | No |
| 74 | 80315 | CPEB4 | 1053 | -0.448 | 0.2383 | No |
| 75 | 4773 | NFATC2 | 1085 | -0.465 | 0.2288 | No |
| 76 | 9939 | RBM8A | 1093 | -0.474 | 0.2375 | No |
Table: GSEA details [plain text format]

  

Fig 2: GO\_RIBONUCLEOPROTEIN\_COMPLEX      
 Blue-Pink O' Gram in the Space of the Analyzed GeneSet

  

Fig 3: GO\_RIBONUCLEOPROTEIN\_COMPLEX: Random ES distribution      
 Gene set null distribution of ES for **GO\_RIBONUCLEOPROTEIN\_COMPLEX**
